# Understanding the Effect of Ionic Liquid–Mediated Solvent Engineering on the Kinetics and Thermodynamic Stability of Phenylalanine Ammonia-Lyase

**DOI:** 10.1021/acs.jpcb.4c04272

**Published:** 2024-09-13

**Authors:** Pranav Bharadwaj, Avishak Barua, Meena Bisht, Dheeraj Kumar Sarkar, Sagar Biswas, Gregory Franklin, Dibyendu Mondal

**Affiliations:** † Institute of Plant Genetics (IPG), Polish Academy of Sciences, Poznań 60-479, Poland; ‡ Centre for Nano and Material Sciences, Jain (Deemed-to-be University), Jain Global Campus, Bangalore, Karnataka 562112, India; § Department of Chemistry, Sri Venkateswara College, University of Delhi, New Delhi, Dhaula Kuan 110021, India; ∥ Tata Institute of Fundamental Research, Hyderabad 50046, India

## Abstract

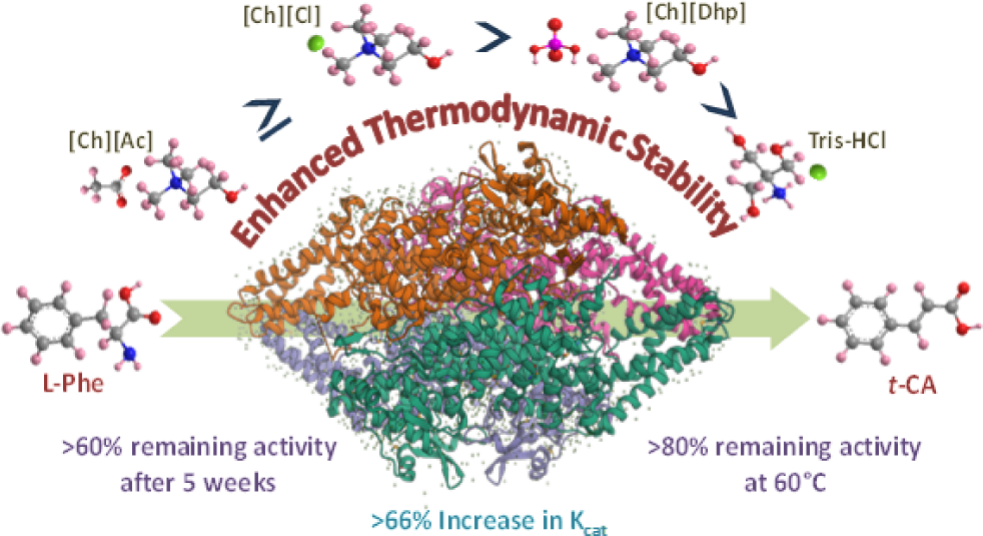

Phenylalanine ammonia-lyase (PAL) plays a central role in the phenylpropanoid pathway and in the treatment of phenylketonuria. However, the integration of PAL into sustainable industrial biocatalysis is hampered by its instability under harsh conditions. This study demonstrates that ionic liquid (IL)–assisted solvent (Tris-HCl buffer) engineering enables improvement of the reaction kinetics and thermodynamic stability of *Rhodotorula glutinis*PAL (*Rg*PAL) under various stresses. Under optimized conditions, a 66.2% higher K_cat_ value, >60% remaining activity after 5 weeks of storage at room temperature, and >80% activity of *Rg*PAL after incubation at 60 °C for 1 h were obtained in the [Ch][Ac]-blended Tris-HCl solvent compared to pristine Tris-HCl. The spectroscopic and molecular docking results suggest that the higher extent of hydration and the soft interactions complemented by the ILs with the D-chain residues of *Rg*PAL jointly contributed to achieving more stable and active conformations of *Rg*PAL. The enzyme showed a higher melting temperature (*T*_m_) in ILs+Tris-HCl compared to that in pristine Tris-HCl, with less change in enthalpy (Δ*H*_fu_) and entropy (Δ*S*_fu_) of unfolding. Overall, IL-mediated solvent engineering alters the microenvironment of *Rg*PAL and allows the development of a robust PAL-based biocatalytic system.

## 1 Introduction

Phenylalanine ammonia-lyase (PAL) is found across living organisms and is known to catalyze the conversion of l-phenylalanine (l-Phe) to *trans*-cinnamic acid.^1^ The enzyme also exhibits bisubstrate specificity for the conversion of l-tyrosine together with its physiological substrate l-Phe and plays a key role in protein synthesis, neurotransmission, and cognitive processes and serves as a precursor for pharmaceutically important compounds.^2,3^ PAL initiates the phenylpropanoid pathway through which various secondary metabolites are biosynthesized in plants for defense against biotic and abiotic stressors.^4−9^ Due to its innate ability to reverse the direction of the deamination reaction by varying physiological and substrate conditions, the use of PAL has been extended to the large-scale chemoenzymatic synthesis of enantiomerically pure l-Phe and a calorie-free artificial sweetener, aspartame.^10^ In medicine, PAL is administered orally to treat an autosomal recessive disorder, phenylketonuria, a condition in which the enzyme phenylalanine hydroxylase loses its ability to convert l-Phe to tyrosine.^11,12^

Despite its significant benefits, the enzyme exhibits lower specific activity and instability under harsh conditions, prompting researchers to improve the efficacy of PAL.^12−18^ Several avenues have been explored in this direction, such as directed evolution,^13^ the permeabilization process,^14^ the use of elicitors (such as amino acids, β-cyclodextrins, and glucuronidase),^15^ γ radiation–mediated targeted site mutagenesis,^16^ the cross-linked enzyme aggregation approach,^17^ immobilization on functional biopolymers,^18^ and conjugation with silk protein.^12^ With the exception of PAL from *Anabaena variabilis* (*Av*PAL), which was developed by directed evolution,^13^ most existing methods focused on increasing PAL concentration in biological systems rather than manipulating the microenvironment of the enzyme to achieve a higher turnover. In addition to biological activity, the stability of PAL is also important for its function under physiological stress conditions over a prolonged period. The stability strategies described so far are limited to directed evolution under extreme conditions. However, a major hurdle for the real-time application of such approaches is the lack of fundamental characterization of the protein and the complexity of sample preparation. Alternatively, the solvent manipulation strategy offers a simple, more environmentally friendly, and sustainable approach that involves simultaneous surface modification and stabilization by green, neoteric solvents.^19−22^ Ionic liquid (IL)–mediated solvent engineering is shown to be promising in perovskite solar cell applications.^23^ However, there is no report so far on the suitability of the solvent engineering approach in manipulating the efficacy of PAL. Therefore, in the present study, a solvent engineering strategy was investigated for a thorough profiling of *Rhodotorula glutinis* PAL (*Rg*PAL) activity and stability in IL-mediated engineered buffer systems. Cholinium-based protein-friendly ionic liquids (ILs), namely cholinium dihydrogen phosphate ([Ch][Dhp]), cholinium chloride ([Ch][Cl]), and cholinium acetate ([Ch][Ac]) were employed in this study. *Rg*PAL was selected for the present work as it exhibits superior catalytic efficiency compared to PAL from most other fungal species and represents a meaningful comparison with the only existing literature using [BMIM]-based ILs as a medium for PAL.^24^ Detailed kinetic and thermodynamic studies using various spectroscopic and in silico approaches were also discussed to understand the suitability of cholinium ILs for engineering the buffer systems envisaging sustainable packaging of *Rg*PAL with enhanced stability and improved kinetics and to present the development of a robust industrial PAL-based biocatalyst.

## 2 Experimental Section

### 2.1 Materials and Methods

*Rg*PAL (CAS: 9024–28–6; Catalog no: P1016–10UN), l-Phe and *trans*-cinnamic acid (*t*-CA) were obtained from Sigma Aldrich. [Ch][Cl] ≥98%, was purchased from Alfa Aesar. [Ch][Ac] >99% and [Ch][Dhp] >98% were purchased from IoliTec, Germany. Tris-HCl buffer, pH 8.5 (200 mM), was used as a reference solvent for preparing the *Rg*PAL solution and IL solutions in double-distilled water. All other chemicals used were of analytical grade.

### 2.2 Activity of *Rg*PAL in the Presence of IL- Mediated Engineered Solvents

The activity of *Rg*PAL in the absence and presence of various concentrations of cholinium-based ILs was recorded in Tris-HCl using a Shimadzu UV-1900i spectrophotometer with a quartz cuvette of 1 cm path length. *Rg*PAL activity was assayed according to the previously reported method with slight modifications.^25^ Briefly, 0.5 μL/mL of the enzyme was equilibrated in 200 mM Tris-HCl buffer solution (pH 8.5), leading to a final concentration of 50 nM in the reaction mixture. This solvent system is taken as the reference to compare the activity and stability of the enzyme with respect to IL+Tris-HCl systems. Similarly, final reaction media of 1 mL containing 10–150 mM ILs were also prepared in the 200 mM Tris-HCl buffer, and the same amount of enzyme was incubated at 25 °C for 1 h. After the interaction, 500 μM of substrate and l-Phenylalanine were added and incubated at 37 °C for 60 min. The change in absorbance was monitored at 270 nm using the kinetic mode. The slope of the curve obtained after plotting time v/s absorbance (270 nm) was analyzed further, and the relative activity of *Rg*PAL was calculated as per the following equation:
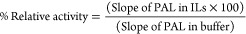


Thus, the activity of *Rg*PAL in 200 mM Tris-HCl (pH 8.5) at 37 °C is taken as 100%, and the activity profiles of all the systems are calculated relative to it. Similarly, for long-term studies, the enzyme was incubated in Tris-HCl and an optimized concentration of ILs+Tris-HCl for 5 weeks at 25 °C, after which the activity assay was carried out and the relative activity was calculated. The effect of temperature on the activity of *Rg*PAL was also performed in the presence of different ILs by incubating the *Rg*PAL sample in an aqueous solution of ILs for 1 h at 60 °C, followed by measuring the enzyme activity. In this case, the remaining activity was calculated at 60 °C by considering the activity of each solvent system at 37 °C as 100%.

### 2.3 Enzyme Kinetics of *Rg*PAL in the Presence of IL-Mediated Engineered Solvents

To study the kinetic parameters of *Rg*PAL, Tris-HCl and the optimized concentrations of individual ILs in Tris-HCl were used. After the initial incubation of *Rg*PAL with solvent systems, varying concentrations of l-Phe substrate were added in separate reaction mixtures, and the concentration was increased until the reaction rate saturated. For this, the concentration range of 10–1750 μM was chosen, and the reaction was monitored for 15 min. The activity of *Rg*PAL in this case is represented in terms of velocity (V), which represents the production of *t*-CA from the substrate by the enzyme per unit of time. The amount of *t*-CA produced is calculated with the help of molar absorption coefficient (ε) of *t*-CA at 270 nm obtained by constructing a standard absorbance (at 270 nm) v/s concentration of *t*-CA curve obeying the Beer–Lambert’s law. To obtain kinetic parameters such as *V*_max_, *K*_m_, and *K*_cat_, Michaelis–Menten (V vs [S]) and Lineweaver–Burk (1/V vs 1/[S]) plots were constructed. The Michaelis–Menten curve gives a typical hyperbolic curve, which is fitted using “Michaelis Menten” function in the “Enzyme kinetics” category, under nonlinear fitting analysis of Origin 2021 software to directly obtain *K*_m_ and *V*_max_. When we fit the curve with the mentioned function, the results are displayed in the following equation:
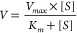


Similarly, the Lineweaver–Burk plot gives a straight line in which the Y-intercept provides the value of 1/*V*_max_, and the slope denotes *K*_m_/*V*_max_ as per the equation appended below:
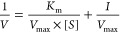


Where, V represents the velocity of the reaction at the given substrate concentration [*S*]; *V*_max_ is the maximum reaction rate, and *K*_m_ represents the Michaelis–Menten constant.

Furthermore, the turnover number *K*_cat_ is calculated by the relation, , where [*E*] represents the concentration of *Rg*PAL, which is 50 nM in all the cases.

### 2.4 UV–visible Second-Derivative Spectra

To characterize solvent-induced structural changes in *Rg*PAL, UV–vis was recorded in the range of 200–800 nm, and structural changes were analyzed in the range of 250–300 nm after the attainment of sample equilibrium. In this case, to get a better qualitative resolution, the concentration of protein was taken to be 5 μL/mL, and the spectra were recorded in a cuvette of 1 cm path length by varying the concentrations of ILs. In this case, the interactions and sampling were done similar to those in activity studies, with the only difference being the absence of the substrate. After the spectra were recorded, they were mathematically transformed into second-derivative spectra using the Origin 2021 software. For both tyrosine and tryptophan residues, sets of minima and maxima were obtained around 280–285 and 290–295 nm, respectively. To study the changes in the environment of *Rg*PAL, the ratio of difference between their individual peaks was calculated as follows.^26^
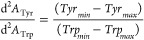


Where Tyr_min_ and Tyr_max_ are the negative and positive d^2^*A*/dλ^2^ peak values in the region of 280–285 nm respectively; and Trp_min_ and Trp_max_ are the negative and positive d^2^*A*/dλ^2^ peak values in the region of 290–295 nm, respectively.

### 2.5 DLS Measurements

Dynamic light scattering (DLS) was carried out using an Anton Paar Litesizer 500 instrument using a cuvette of 1 cm path length. Sampling was done similar to that of CD analysis, and before analyzing the sample, incubated media were filtered through syringe filters having 0.02 μm pore size. With the background as water, the DLS program was run at 25 °C in protein mode with a maximum of 60 cycles, and the options namely quality, filter, focus, and measurement angle were set in the automatic mode. Three independent samplings were done for each solvent system, and the average of the data with standard deviation was plotted.

### 2.6 Thermal Stability of *Rg*PAL in the Presence of IL-Engineered Solvents

The stability of *Rg*PAL in the presence of cholinium-based ILs+Tris-HCl at different temperatures was studied by circular dichroism (CD) spectroscopy for which a Jasco-1500 spectrophotometer equipped with a Peltier system for temperature control was utilized. The spectra of *Rg*PAL were acquired with quartz cuvettes of path length 1 mm (JASCO type J/21) and a final concentration of 2 μL/mL of enzyme in all cases. Each sample spectrum was obtained by subtracting the appropriate blank sample from the experimental spectrum and was collected by averaging three spectra. After the samples were pre-equilibrated, CD spectra in the range of 190–250 nm were taken from 25 to 90 °C with a heating rate of 1 °C min^–1^, nitrogen flow rate of 4 L min^–1^, data interval of 5 °C, and 100 nm min^–1^ scan speed. Each CD spectrum represents an average of three scans followed by 25 points smoothening, and the baseline of the CD spectrum was corrected by subtracting the corresponding reference samples. To determine the melting temperature (*T*_m_), the unfolding pattern following a two-state model was considered, and the corresponding fraction of the unfolded state (*f*_U_) and native state (*f*_N_) was calculated using the following relations.
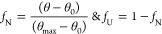


Where, θ is the CD signal of the corresponding temperature at 222 nm; θ_0_ is the signal of minimum intensity relating to completely denatured state, and θ_max_ is the signal with maximum magnitude (in the negative axis) representing the native state of the enzyme. The plot of *f*_U_ v/s temperature gives a sigmoidal curve, the derivative of which gives a single peak corresponding to the melting temperature (*T*_m_) of *Rg*PAL for a given system. Thermodynamic parameters such as Gibbs free energy, enthalpy, and entropy of unfolding are calculated by first evaluating the equilibrium constant K at all temperatures as follows.
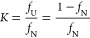

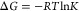


Furthermore, a plot of ln *K* vs 1/T is created, and the linear region is fitted to obtain the intercept and slope, which provide the values of enthalpy (ΔH) and entropy of unfolding (ΔS) as follows:
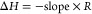

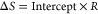


where R is the universal gas constant = 1.987 × 10^–3^ kcalK^–1^ mol^–1^

### 2.7 Molecular Docking

The homology modeling of the structure of *Rg*PAL was derived from the sequence of *Rhodotorula glutinis* (GenBank accession no. AHB63479), using the crystal structure of the template protein with PDB ID 1Y2M, as implemented previously by Hendrikse et al. 2020.^27^ The Swiss model program was used for homology modeling.^28^ The modeled structure was repaired for the correction of improper clashes or torsions of any residues using the Foldx program.^29^ We investigated the binding affinities of Tris-HCl, [Ch][Cl], [Ch][Ac], and [Ch][Dhp] solvents for which Tris-HCl, acetate, choline, and dihydrogen phosphate ions were individually docked using the AutoDock Vina program in *Rg*PAL enzymes.^30^ Blind docking was performed, keeping a uniform box size of 37 × 26 × 27 nm across all replicates and for each molecule, and the docking scores and top positions of the solvents were inferred. A total of the top 10 poses were assessed, and the highest affinity positions are taken from all three replicates.

## 3 Results and Discussion

### 3.1 Relative Activity and Reaction Kinetics of *Rg*PAL in Various IL-Mediated Engineered Solvents

**Figure 1:**
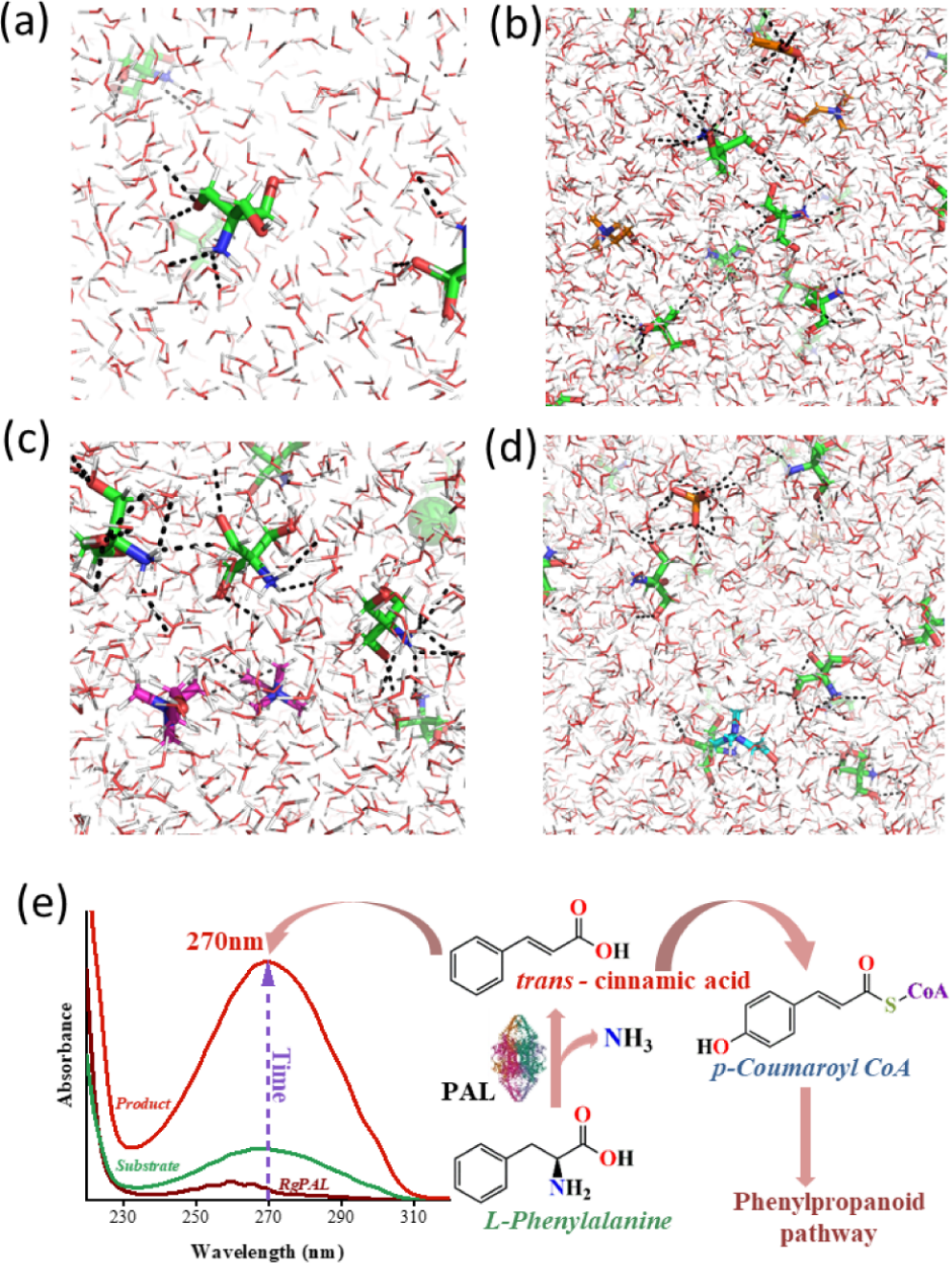
Polar interaction of Tris-HCl (a), Tris-HCl+[Ch][Ac] (b), Tris-HCl+[Ch][Cl] (c), and Tris-HCl+[Ch][DhP] (d). Tris-HCl, [Ch][Ac], [Ch][Cl], and [Ch][DhP] are shown in green, orange, magenta, and cyan, respectively. The box dimension of 10 × 10 × 10 nm^3^ was prepared using packmol package. (e) Representation of PAL activity with l-Phe as the substrate and its corresponding activity assay using UV–vis spectroscopy.

The molecular structure of 200 mM Tris-HCl buffer in H_2_O within box dimension of 10 × 10 × 10 nm^3^ is shown in Figure 1a. The addition of 50 mM [Ch][Ac], [Ch][Cl], and [Ch][DhP], respectively, to 200 mM Tris-HCl enhances intra- and intermolecular polar interactions among solutes in different molecular systems of Tris-HCl, ILs, and H_2_O (Figure 1b–d).

The effect of such simple solvent engineering on the catalytic activity of *Rg*PAL was studied next. When the reaction mixture containing *Rg*PAL is introduced with the substrate l-Phe, it forms an adduct with the catalytic site of the enzyme (N-MIO), which further leads the reaction to undergo deamination through either E1cB, E1, E2, or FC mechanisms to give *t*-CA,^31^ which is subsequently involved in the phenylpropanoid pathway via the *p*-coumaroyl CoA intermediate (Figure 1e). The product *t*-CA gave a characteristic peak at 270 nm, and the activity of *Rg*PAL was studied using the rate of formation of *t*-CA using UV–vis spectroscopy (Figures 1e and S1).

**Figure 2:**
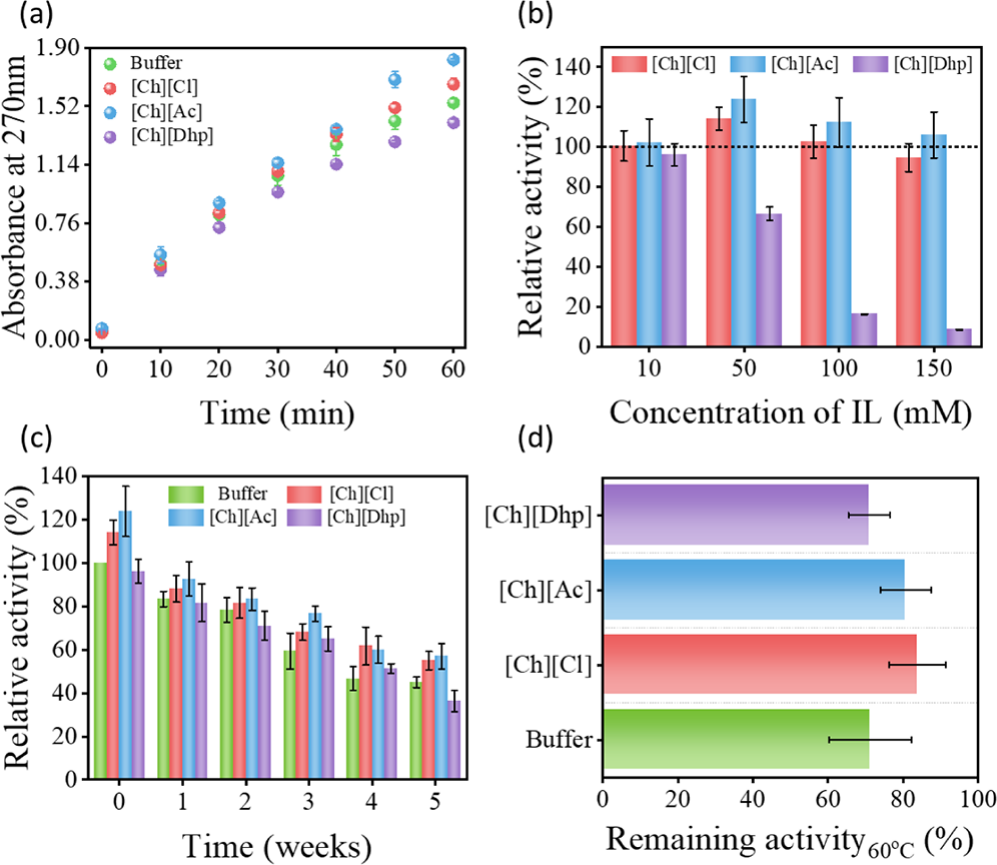
(a) Time-dependent variation of absorbance at 270 nm of *trans*-cinnamic acid in buffer (Tris-HCl) and ILs+Tris-HCl at optimized concentrations. (b) Relative activity of *Rg*PAL at different concentrations of ILs in Tris-HCl, (c) Relative activity of *Rg*PAL in buffer, 10 mM of [Ch][Dhp], and 50 mM of [Ch][Cl] and [Ch][Ac] solutions after incubation for 0–5 weeks at room temperature,and (d) remaining activity of *Rg*PAL in buffer and IL media after incubation at 60 °C for 1 h.

The progress of the reaction was monitored by absorbance versus time (Figure 2a), which formed a straight line up to 45 min. After that, it started to saturate, indicating that 45 min is the optimal incubation time for measuring the activity of *Rg*PAL. This result agrees well with previous studies in which the optimal PAL activity was achieved after an incubation time of 30–45 min.^24^ The relative activity of *Rg*PAL in different IL-modified Tris-HCl was compared by considering 100% activity in pristine Tris-HCl buffer (pH 8.5). Figure 2b shows that different concentrations (10–150 mM) of [Ch][Ac] and [Ch][Cl] affect the RgPAL activity. At 50 mM, [Ch][Ac] and [Ch][Cl] increased the activity by 1.23-fold and 1.14-fold, respectively, compared to RgPAL in Tris-HCl buffer. Notably, RgPAL activity was lower at 10 mM for both [Ch][Ac] and [Ch][Cl], peaked at 50 mM, and then decreased as the concentration increased to 100 mM and 150 mM. However, no significant difference in activity was observed between [Ch][Ac] and [Ch][Cl] at 50 mM and 100 mM concentrations. In contrast, [Ch][Dhp], which is known for its protein stabilization, showed a relative activity of 96% at 10 mM compared to the buffer system. However, increasing the [Ch][Dhp] concentration from 10 mM to 50 mM resulted in a further decrease in activity, and at 150 mM, *Rg*PAL activity was nearly absent. The reasons for this behavior with [Ch][Dhp] are discussed in Section 3.2. It is important to note that the activity of *Rg*PAL in protein-friendly IL-mediated engineered solvent is significantly higher than the previous report, which found a relative activity of 86% with the best optimized system [BMIM][PF_6_].^24^

**Figure 3:**
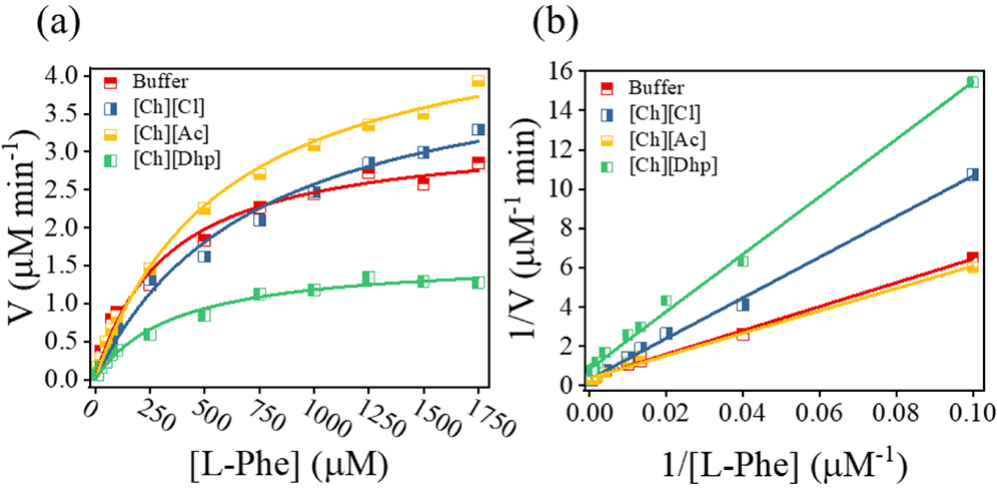
Kinetic studies of *Rg*PAL in buffer and IL media are represented using (a) Michaelis–Menten and (b) Lineweaver–Burk plots.

*Rg*PAL is a sensitive enzyme that is normally stored at −20 °C to maintain its structural integrity in the long term. Previous studies show the potential of [Ch][Cl] and [Ch][Dhp] as long-term storage media for antibodies.^32^ Accordingly, the effect of these ILs on the activity of *Rg*PAL was investigated in engineered solvents when stored at room temperature for up to 5 weeks (Figure 2c). As shown in Figure 1e, > 50% PAL is deactivated after 4 weeks of storage in a buffer system. However, in the presence of [Ch][Ac] and [Ch]Cl] >60% of the activity is retained even after 5 weeks of storage under ambient conditions. Thus, engineering Tris-HCl buffer with these two ILs not only led to an increased activity of *Rg*PAL, but is also suitable for long-term packaging of the enzyme at room temperature. Furthermore, the activity of *Rg*PAL was investigated under thermal stress after 1 h of incubation at 60 °C in optimized concentrations of ILs+buffer systems. The above temperature was selected based on the previous study on PAL from *R. glutinis* and related species.^33^Figure 2d shows the percentage of residual activity at high temperatures in different solvent systems. While the buffer system at 60 °C retained 70% of its original activity, which is close to the previous report,^33^ [Ch][Ac]- and [Ch][Cl]-modified solvents showed retention of ∼80% activity, proving that they are better systems to protect *Rg*PAL from thermal deactivation. Considering the beneficial effects of solvent engineering by cholinium ILs toward the catalytic activity of *Rg*PAL under various stress conditions, a study was further conducted to investigate the enzyme kinetics in engineered solvent systems. The reaction rate was monitored in a time frame of 15 min before saturation by changing the concentration of l-Phe from 10 to 1750 μM. Our results show that *Rg*PAL follows the Michaelis–Menten and Lineweaver–Burk models in all cases, with an R^2^ fit higher than 0.98 and 0.99, respectively (Figure 3a,b).

The corresponding values for the maximum reaction rate (*V*_max_), Michaelis–Menten constant (*K*_m_), and turnover number (*K*_cat_) resulting from the fit of Figure 2b are summarized in Table 1. Both *V*_max_ and *K*_cat_ follow a trend similar to that of the relative activity studies. Remarkably, a 66.2% higher *K*_cat_ value was obtained in the [Ch][Ac]-added Tris-HCl system compared to that in only Tris-HCl. The *K*_m_ values were in the order of Tris-HCl < [Ch][Dhp] < [Ch][Ac] < [Ch][Cl], indicating that the buffer media have a higher substrate affinity than the IL-engineered Tris-HCl media. Since the *V*_max_ of [Ch][Dhp] decreases with a slight increase in *K*_m_, this is one of the cases of kinetic inhibition. Based on this pattern and the molecular dissimilarities between l-phe and [Ch][Dhp], it could be an allosteric inhibition. In the case of the other two ILs, both *K*_m_ and *V*_max_ were increased, suggesting that [Ch][Ac] and [Ch][Cl] ILs act as effectors.^33,34^ These modulations of the kinetic properties of the enzyme are caused by conformational changes due to soft interactions such as H bonding and electrostatic interactions between ion pairs of ILs and amino acid residues of the protein.

**Table 1:** Obtained Values of *K*_m_, *V*_max_, and *K*_cat_ from Lineweaver–Burk Plots

	*V*_max_ (μM min^–1^)	*K*_m_ (μM)	*K*_cat_ (min^–1^)
Buffer	2.57 ± 0.16	156.20 ± 5.56	51.55 ± 3.2
[Ch][Cl]	3.50 ± 0.32	415.79 ± 10.59	70.13 ± 6.94
[Ch][Ac]	4.28 ± 0.13	409.59 ± 19.91	85.63 ± 2.6
[Ch][Dhp]	1.20 ± 0.12	177.18 ± 4.78	24.16 ± 2.4

### 3.2 Molecular Docking of *Rg*PAL in Various Engineered Solvents

**Figure 4:**
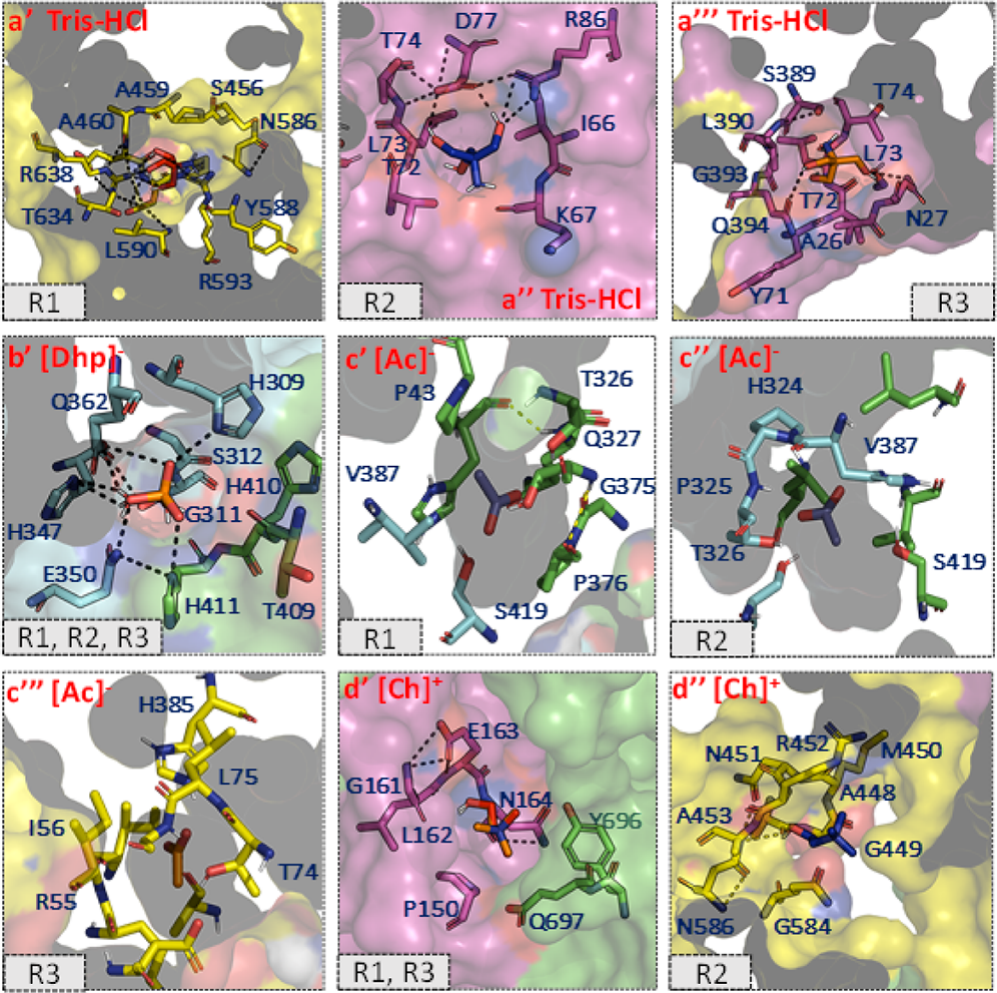
Top docked poses of (a’–a’’’) Tris HCl, (b’) dihydrogen phosphate [Dhp]^−^, (c’–c’’’) acetate [Ac]^−^, and (d’–d’’) choline [Ch]^+^ on high-affinity cavities of *Rg*PAL from replicate1 (R1), replicate 2 (R2), and replicate 3 (R3). Polar interactions and residues forming the cavity pocket of docked poses are depicted as stick representations. Chains A, B, C, and D are shown in green, cyan, magenta, and yellow, respectively.

To gain further insights into the binding affinity between protein and ILs, in silico docking studies were performed in which Tris-HCl^–^, [Ac]^−^, [Ch]^+^, and [Dhp]^−^ ions were individually docked using the AutoDock Vina program.^30^ The poses with the highest affinity docking to three of the replicates were determined by blind docking in three replicates for the systems Tris-HCl, [Ch]^+^, [Ac]^−^, and [Dhp]^−^. These binding sites are summarized in Figure S2, and the corresponding docking scores are summarized in Tables S1–S4. Tris-HCl exhibited the highest overall affinity of −4.6 to −4.3 kcal/mol (Table S1). Three binding pockets were determined from the top scores for the solvent Tris-HCl (Figures 4a’–a’’’ and S2a). In the binding pockets, Arg593, Arg638 (R1); Asp77, Arg86 (R2); Asn27 and Gln394 (R3) shared polar contacts with Tris-HCl. Interestingly, all three replicates docked to the same cavity for [Dhp]^−^ (Figures 4b’ and S2b) and formed polar interactions with His309, Glu350, and Gln362, emphasizing the potential of this position and the residues forming the binding cavity for stable interactions. The binding cavities of [Ac]^−^ and [Ch]^+^ shown above are depicted in Figure 4c’–c’’’,d’–d’’, respectively, and fewer polar interactions were observed in both cases. The [Ch]^+^ counterpart showed better polar interaction with the pockets; however, the preferred positions R2 of [Ch]^+^ were similar to those of R1 of Tris-HCl, as shown in Figures 4a’,d’’ and S2b. Overall, the region of chain D (∼ residues 450–650) can be considered as a potential site for *Rg*PAL since most of the compounds and ion pairs were docked at this position. Thus, by evaluating the favorable binding energy, the solvent systems can be classified as Tris-HCl > [Dhp]^−^ > [Ch]^+^ > [Ac]^−^. Although the affinity of Tris-HCl is higher than that of ILs, the binding sites of Tris-HCl are different from those of ILs. Since the binding sites of ILs are close to the loci (Figure S2b), the trend of activity is inversely proportional to the corresponding binding affinities of the anionic counterpart of the ILs studied. The conformational changes of *Rg*PAL due to the soft interactions between the enzyme and ILs were further investigated by various spectroscopic characterizations.

### 3.3 UV–Vis, Second-Derivative UV, DLS, and CD Spectra of *Rg*PAL in Various IL-Mediated Engineered Solvents

**Figure 5:**
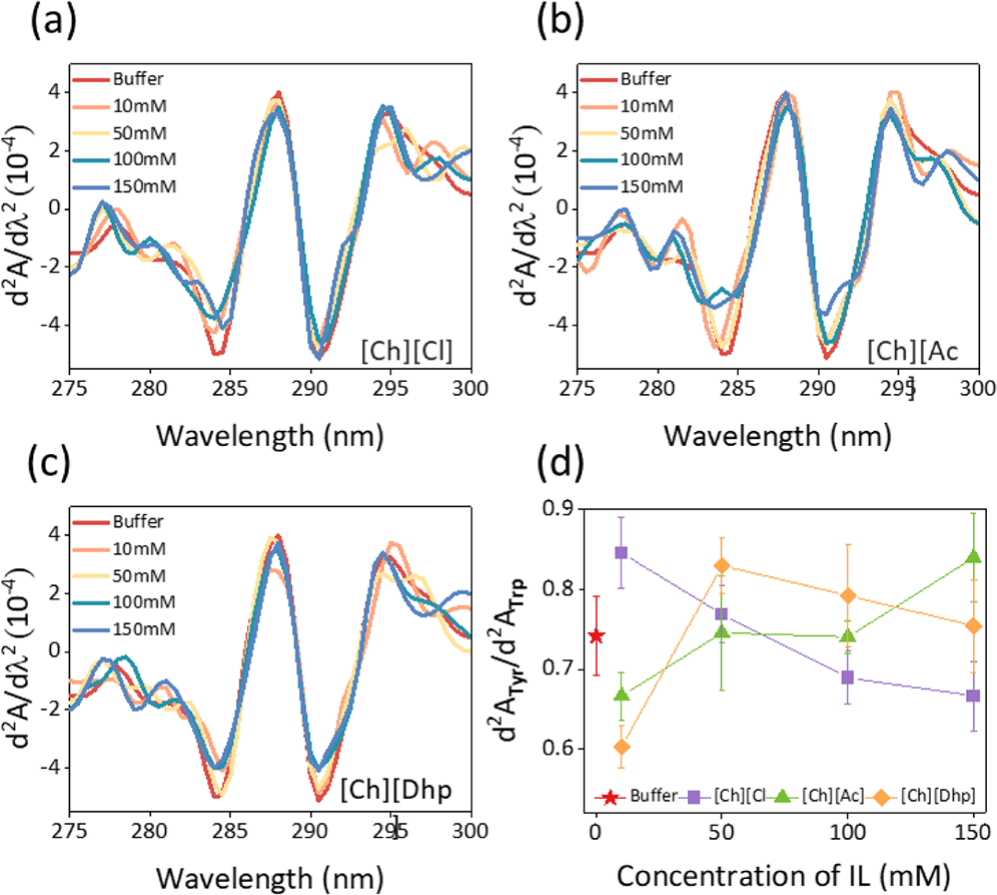
(a–c) Second-derivative UV–vis spectra of *Rg*PAL in buffer (Tris-HCl) and IL media and (d) corresponding peak–peak distance ratios of tyrosine to tryptophan residues depicting the structural changes brought about bychanging concentrations of ILs.

When an enzyme unfolds, its aromatic amino acid residues are exposed by solvation of the surrounding environment and can be characterized both qualitatively and quantitatively by UV–vis spectroscopy.^26^*Rg*PAL shows a broad peak around 280 nm consisting of two peaks generated by the chromophores of tyrosine (ca. 285 nm) and tryptophan (ca. 295 nm). When the IL concentration was increased from 10 to 150 mM, a hyperchromic shift was observed in all cases, which was particularly pronounced in the case of [Ch][Ac] (Figure S3). In all the cases, soon after the addition of ILs, the intensity decreases compared to the buffer, but with increasing IL concentration, in all ILs, a hyperchromic shift was observed (Figure S3). The extent of hydration can be further analyzed using the d^2^*A*/dλ^2^ values at 285 nm (Figure 5). As hydration around the protein increases, the intensity of the peak increases toward a more negative value.^35^ In all cases, the ILs are expected to have a lower extent of hydration than buffers as the solutions of the ILs were prepared in the buffer itself, leading to a decrease in the water activity of the media. Figure 5a–c shows that the hydration trend of ILs follows the order [Ch][Dhp] > [Ch][Ac] > [Ch][Cl], which is also confirmed by DLS studies (Figure S4). It was found that PAL in Tris-Hcl has a hydrodynamic radius (*D*_h_) of ∼14.3 nm, and this value increases in the presence of ILs and is in the range of 15.65–16 nm (Figure S8b). When ILs are introduced into a solvent media, they not only change the hydration sphere around the protein but are also known to interact directly with the proteins, which in turn increases the *D*_h_ value marginally.^20^ Therefore, the DLS results can be attributed to the combination of RgPAL-IL soft interactions and the hydration effect. In the latter case, the microenvironment of the enzyme is usually altered.

**Figure 6:**
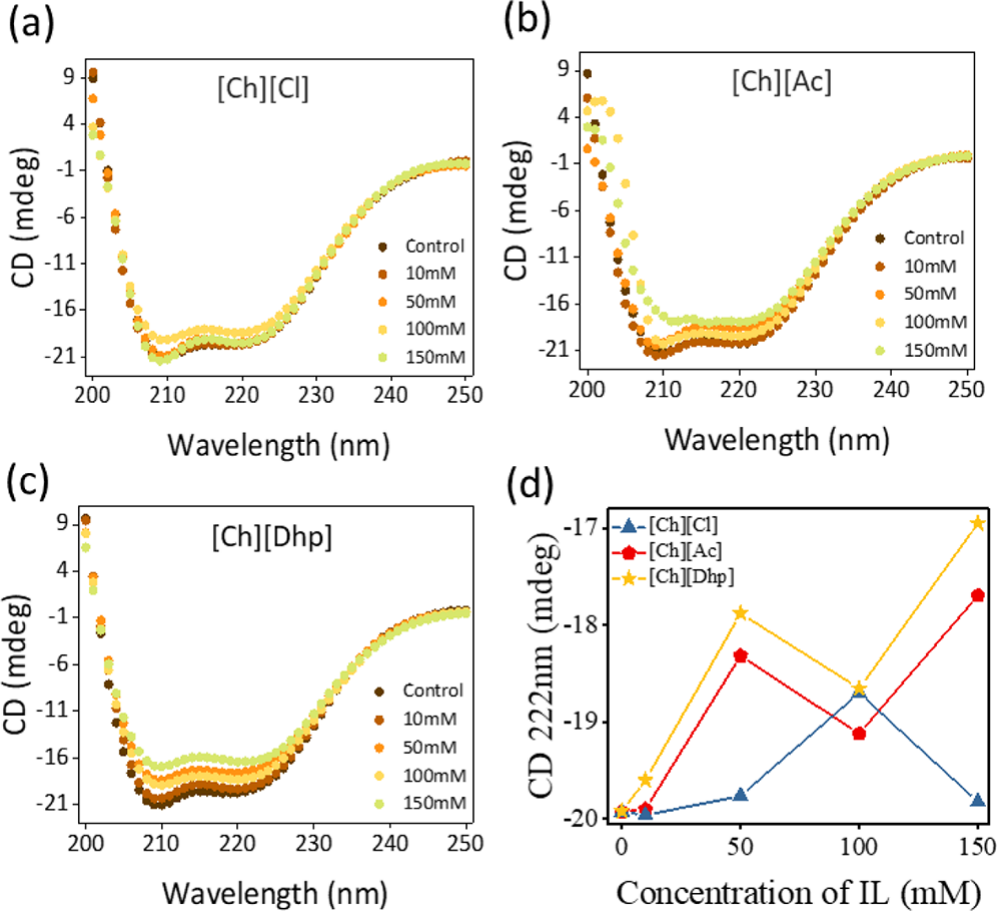
Far-UV circular dichroism spectra of *Rg*PAL with varying concentrations of (a)[Ch][Cl], (b)[Ch][Ac] and (c) [Ch][Dhp] ILs, respectively, in Tris-HCl. (d) CD values at various concentrations of ILs in Tris-HCl. In all the cases, spectra were recorded with 2 μL/mL concentration of *Rg*PAL in a quartz cuvette having 1 mm path length.

From the second-derivative spectra, the peak-to-peak ratio (d^2^*A*_Tyr_/d^2^*A*_Trp_) was calculated following previous reports.^26^ Upon the addition of ILs ([Ch][Ac] and [Ch][Dhp]) to buffer media at a concentration of 10 mM, a decrease in d^2^*A*_Tyr_/d^2^*A*_Trp_ values was observed compared to Tris-HCl, indicating the compactness of the aromatic residues (Figure 5d). As the concentration of these ILs increased, the d^2^A_Tyr_/d^2^A_Trp_ values increased, suggesting that the aromatic residues are exposed due to the polarity of the solvent environment. In the case of [Ch][Cl], a different trend was observed where the d^2^*A*_Tyr_/d^2^*A*_Trp_ values decreased with an increasing concentration of [Ch][Cl]. This suggests that apart from the solvent nature, IL–protein interactions are prevalent at higher concentrations, leading to conformational changes of the enzyme.^26,35^ Regardless of the concentration range, the d^2^*A*_Tyr_/d^2^*A*_Trp_ values in all ILs remain within the limits of the folding conformations. To strengthen this observation, a concentration-dependent CD analysis of *Rg*PAL in ILs+Tris-HCl systems was monitored at 25 °C using far-UV CD spectra in the 200–250 nm range. The far-UV CD spectra (Figure 6a–d) clearly showed that PAL is associated with a higher content of α-helical secondary structures as the spectra have corresponding characteristic maxima at 209 and 222 nm.^36^ As the concentration of ILs increased, the intensity of the CD curves decreased at these two values, suggesting a decrease in the α-helical content and an increase in the β-sheet. However, these changes were marginal for all ILs across all concentration ranges, and only partial unfolding of *Rg*PAL was observed (Figure 6d).

### 3.4 Thermodynamic Stability of *Rg*PAL in Various IL-Mediated Engineered Solvents

**Figure 7:**
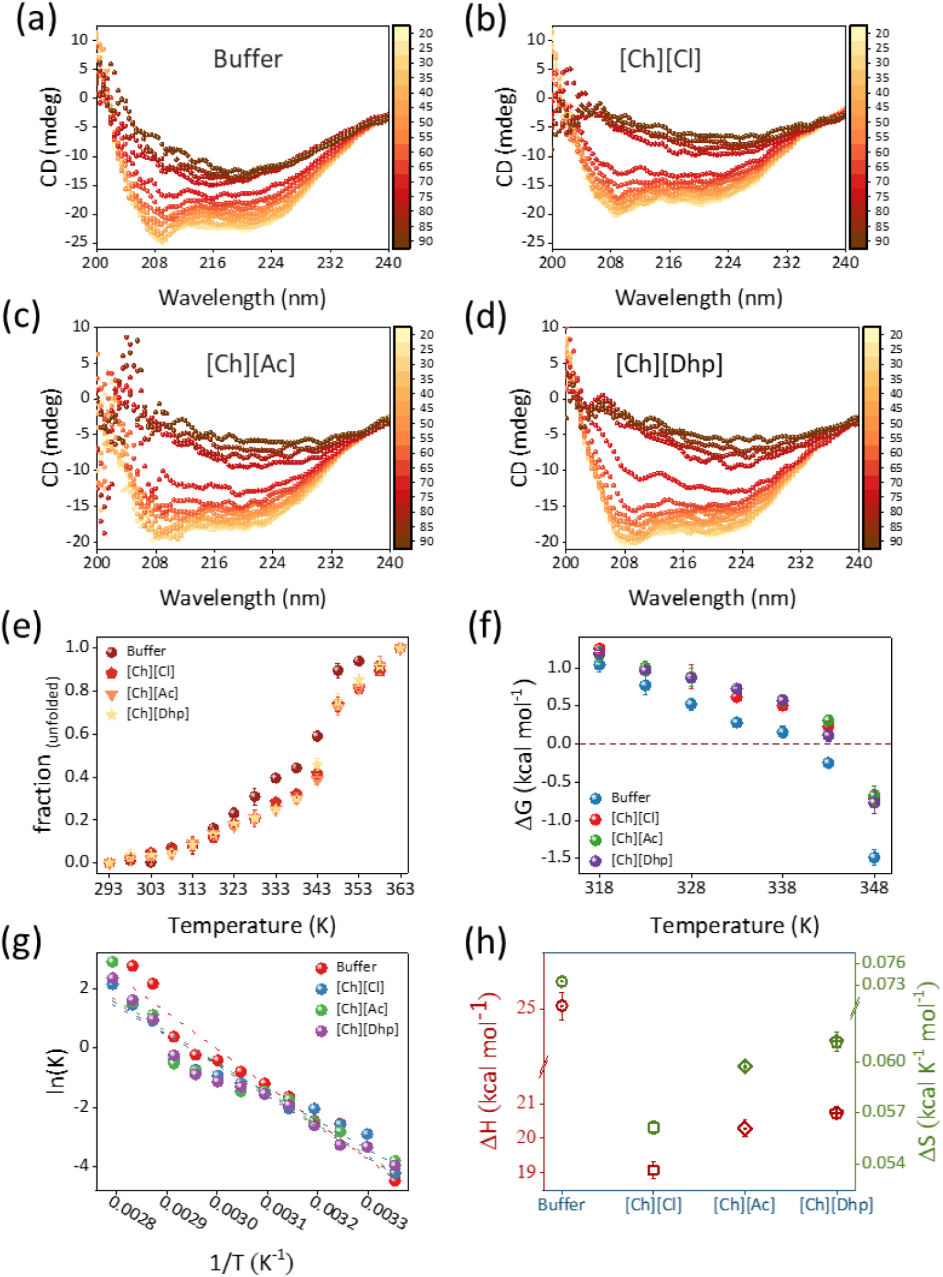
(a–d) Far-UV CD spectra of *Rg*PAL in buffer and ILs as a function of temperature, (e) corresponding sigmoidal curve of temperature-dependent fraction of unfolded enzyme depicting two-state transition. (f) Represents the stability curve of *Rg*PAL followed by (g) vant-Hoff plot leading to (h) the values of change in enthalpy (Δ*H*) and entropy (Δ*S*) of protein unfolding in buffer and IL+Tris-HCl systems.

To further explain the structural stability of RgPAL by the ILs, the thermodynamic parameters were evaluated using a temperature-dependent CD analysis by varying the temperature from 25 °C to temperatures above the melting temperature (*T*_m_) of *Rg*PAL (Figure 7a–d). When the temperature was increased from 25 °C to > *T*_m_, the CD values at 209 and 222 nm decreased in a sigmoidal fashion, inferring that the denaturation of PAL occurs through a decrease in the proportion of the α-helix and a simultaneous increase in the β-sheet and random coils. To analyze the thermodynamic stability, the *T*_m_ values of PAL in each solvent system were calculated by plotting the fraction of unfolded PAL v/s temperature. This results in a sigmoidal curve (Figure 7e), the derivative of which gives the *T*_m_ value.^36^ As can be seen from Figure 7e, the enzyme undergoes two unfolding phases, one in the 30–50 °C range, which is due to H bond breaking and soft interactions, and the other transition takes place around *T*_m_ and leads to denaturation. The *T*_m_ values for *Rg*PAL in Tris-HCl were 69.22 °C, which is consistent with a previous report.^27^ In the presence of all ILs at optimized concentrations, *Rg*PAL showed higher *T*_m_ values such as 73.32 °C, 73.25 °C, and 74.02 °C for [Ch][Cl], [Ch][Dhp], and [Ch][Ac], respectively. Thus, the thermal stability of *Rg*PAL was increased by 4–5 °C in the presence of protein-friendly ILs compared to pristine buffer. Such an increase in the thermal stability of *Rg*PAL in IL+Tris-HCl systems is favored by additional soft interactions such as H bonds and electrostatic interactions between ion pairs of the IL and the amino acid residues of the enzyme. The increased thermal stability of PAL in ILs is underpinned by the stability curve, which is a plot of Δ*G*_fold_ → unfold (Δ*G*_fu_) v/s temperature (Figure 7f). The dotted value Δ*G* = 0 represents the state in which *f*_N_ = *f*_U_. It can be seen from Figure 7f that the stability curve for the buffer system has a slope higher than that of the ILs and thus crosses the value Δ*G* = 0 at a much lower temperature than that of the ILs. This indicates that *Rg*PAL in buffers tends to unfold faster than *Rg*PAL in ILs+Tris-HCl. This observation is further supported by changes in enthalpy (Δ*H*_fu_) and entropy (Δ*S*_fu_) during unfolding (Figure 7g,h and Table S1). The values of Δ*H*_fu_ and Δ*S*_fu_ show a cooperative effect in the enzyme, where the unfolding process is endothermic, and the enzyme absorbs a maximum amount of heat in the final state of unfolding. Thus, assuming that the molar heat capacity of an enzyme is constant throughout the process, the heat absorbed by the enzyme would lead to a disordered state and thus have a positive Δ*S*_fu_ value.^36^ In this context, *Rg*PAL in buffer showed a higher Δ*H*_fu_ (24.5 kcal mol^–1^) and Δ*S*_fu_ (0.074 kcal K^–1^) value compared to all IL engineered buffer systems, indicating that *Rg*PAL in buffer has a higher tendency to transition to a denatured state than the ILs+Tris-HCl systems. Among the ILs, Δ*H*_fu_ and Δ*S*_fu_ were found in the order of [Ch][Dhp] (20.72 kcal mol^–1^ and 0.061 kcal K^–1^) > [Ch][Ac] (20.28 kcal mol^–1^ and 0.059 kcal K^–1^) > [Ch][Cl] (19.07 kcal mol^–1^ and 0.056 kcal K^–1^). In general, the thermodynamic parameters indicate that the thermal stability of *Rg*PAL in different solvent media follows the order [Ch][Ac] ≳ [Ch][Cl] > [Ch][Dhp] > Tris-HCl buffer.

## 4 Conclusion

In summary, detailed kinetic and thermodynamic studies using various spectroscopic and in silico approaches were discussed to understand the suitability of IL-mediated solvent engineering strategy for packaging of *Rg*PAL, envisaging sustainable development of a robust industrial PAL-based biocatalysis. The best IL was [Ch][Ac], which showed a 66% higher Kcat of *Rg*PAL than Tris-HCl at a concentration of 50 mM. Although [Ch][Dhp] is known to increase the efficacy of many proteins, including heme proteins, it had a detrimental effect on the turnover number of *Rg*PAL. Overall, the relative activity followed the trend [Ch][Ac] > [Ch][Cl] > Tris-HCl > [Ch][Dhp]. UV–vis and DLS studies show that all ILs affect the hydration layer of *Rg*PAL and the changes in the microenvironment of the enzyme. However, no unfolding was observed in all IL systems, as confirmed by the d^2^*A*_Tyr_/d^2^*A*_Trp_ values and the concentration-dependent CD spectra. Due to the higher extent of hydration and the additional soft interactions that the ILs make mainly with chain D (∼residues 450–650) of *Rg*PAL, the enzyme achieves more stable and active conformations. Although the activity of *Rg*PAL was relatively lower in [Ch][Dhp], the stability was higher than that in the buffer system. In addition, all ILs showed improved thermodynamic stability of *Rg*PAL with higher *T*_m_, lower Δ*H*_fu_, and less Δ*S*_fu_ compared to the buffer. Overall, solvent engineering by ILs offers an ecofriendly and sustainable biocatalytic media for *Rg*PAL with improved kinetics and increased thermodynamic stability.

## Supporting Information Available

Figures S1–S4 and Tables S1–S5 is available. Figure S1 represent UV–vis spectra of *Rg*PAL activity assay. Figure S2 shows docking poses of *Rg*PAL with various systems. Figure S3 and S4 show UV–vis and hydrodynamic radius of *Rg*PAL, respectively. Table S1–S4 show docking score of *Rg*PAL with various solvents and Figure S5 shows the data of entropy and enthalpy of unfolding (PDF)

## List of Abbreviation:

RgPAL: *Rhodotorula glutinis* Phenylalanine ammonia lyase
l-Phe: l-phenylalanine
*t*-CA: *trans*-cinnamic acid
IL: Ionic liquid
Tris-HCl: Tris(hydroxymethyl)-aminomethan-hydrochloride
[Ch]^+^: Choline
[Cl]^−^: Chloride
[Ac]^−^: Acetate
[Dhp]^−^: Dihydrogen phosphate
Tyr: Tyrosine
Trp: Tryptophan
CD: Circular dichroism
UV–vis: Ultraviolet–visible
