# Supplementary material for: Understanding the Effect of Ionic Liquid–Mediated Solvent Engineering on the Kinetics and Thermodynamic Stability of Phenylalanine Ammonia-Lyase
Source: J Phys Chem B. 2024 Sep 13;128(38):9102–10. doi: 10.1021/acs.jpcb.4c04272 (PMC11440588; doi:10.1021/acs.jpcb.4c04272)
Supplement: Supplementary file 1 — jp4c04272_si_001.pdf [file jp4c04272_si_001.pdf]

Supplementary information

Understanding the Effect of Ionic Liquid Mediated Solvent Engineering on the Kinetics and Thermodynamic Stability of Phenylalanine Ammonia-lyase

Pranav Bharadwaj,[‡,1,2] Avishak Barua,[‡,1] Meena Bisht,[1,3] Dheeraj Kumar Sarkar,[4] Sagar Biswas,[1] Gregory Franklin*[1] and Dibyendu Mondal*[1,2]

[1] Institute of Plant Genetics (IPG), Polish Academy of Sciences, Strzeszyńska 34, 60-479 Poznań, Poland.

[2] Centre for Nano and Material Sciences, Jain (Deemed-to-be University), Jain Global Campus, Kanakapura, Bangalore, Karnataka, India.

[3] Department of Chemistry, Sri Venkateswara College, University of Delhi, Dhaula Kuan, New Delhi 110021, India.

[4] Tata Institute of Fundamental Research, Hyderabad 50046, India.

‡. PB and AB have contributed equally towards this work.

*Corresponding authors, e-mail: GF: fgre@igr.poznan.pl; DM: m.dibyendu@jainuniversity.ac.in; dmon@igr.poznan.pl

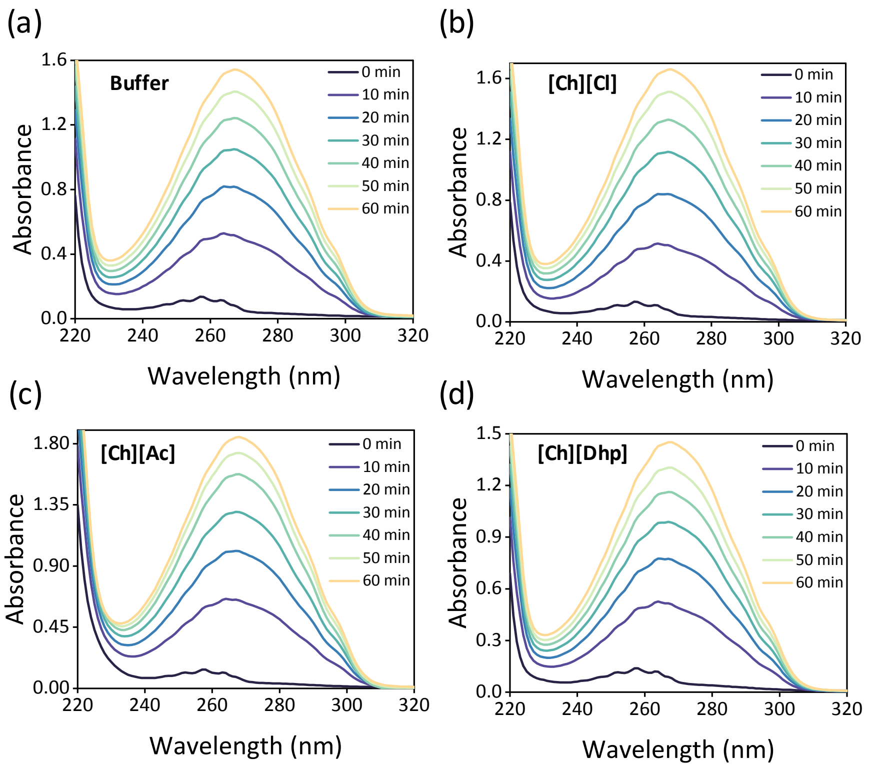

Figure S1. UV-Vis spectra of course of the *Rg*PAL activity assay using in (a)Tris-Hcl buffer and (b-d) ionic liquid media.

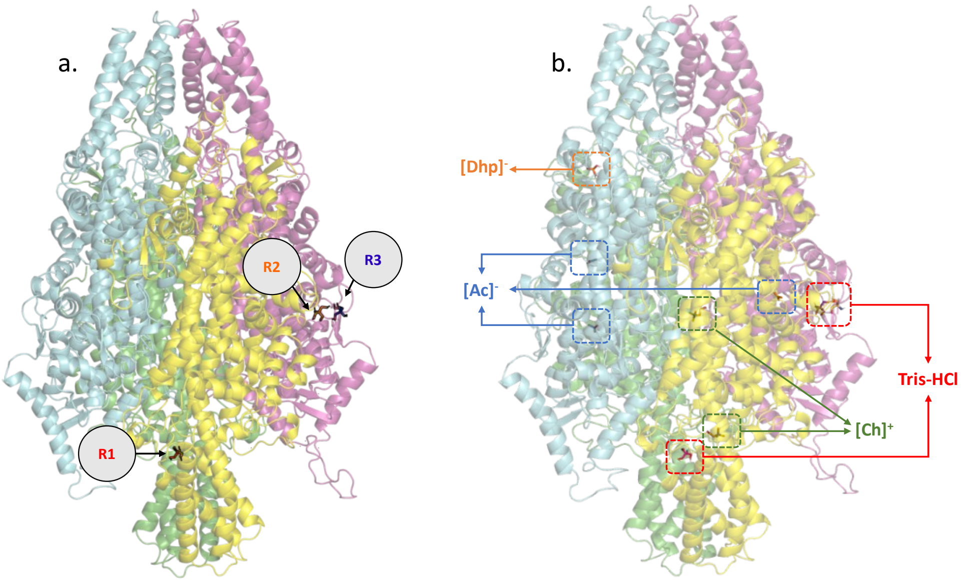

Figure S2. Peptide chains A, B, C and D of *Rg*PAL are shown in green, cyan, magenta and yellow, respectively with (a) top docked poses of Tris-HCl on the high affinity cavities of *Rg*PAL from replicate 1 (red), replicate 2 (blue) and replicate 3 (orange) and (b) Top docked poses of Tris-HCl, dihydrogenphosphate ([Dhp]$^-$), acetate ([Ac]$^-$) and choline ([Ch]$^+$) on the high affinity cavities of *Rg*PAL.

Table S1. Docking score for Tris HCl with respective to the ten poses and the binding affinity scores for replicate 1 (R1-SCORE), replicate 2 (R2-SCORE) and replicate 3 (R3-SCORE). Energy scores are in kcal/mol units.

RANK	NAME	POSE	R1-SCORE	R2-SCORE	R3-SCORE
1	Tris HCl	1	-4.5	-4.3	-4.6
2	Tris HCl	2	-4.3	-4.3	-4.3
3	Tris HCl	3	-4	-4.3	-4.3
4	Tris HCl	4	-3.9	-4.2	-4.1
5	Tris HCl	5	-3.8	-4	-4.1
6	Tris HCl	6	-3.6	-4	-4
7	Tris HCl	7	-3.6	-4	-4
8	Tris HCl	8	-3.6	-4	-3.9
9	Tris HCl	9	-3.6	-4	-3.9
10	Tris HCl	10	-3.6	-3.9	-3.8

Table S2. Docking score for Acetate with respective to the ten poses and the binding affinity scores for replicate 1 (R1-SCORE), replicate 2 (R2-SCORE) and replicate 3 (R3-SCORE).

RANK	NAME	POSE	R1-SCORE	R2-SCORE	R3-SCORE
1	Acetate	1	-3.3	-3.2	-3.3
2	Acetate	2	-3.1	-3	-3.3
3	Acetate	3	-3.1	-3	-3
4	Acetate	4	-3.1	-3	-3
5	Acetate	5	-3	-3	-2.9
6	Acetate	6	-3	-3	-2.9
7	Acetate	7	-2.9	-2.9	-2.9
8	Acetate	8	-2.9	-2.8	-2.8
9	Acetate	9	-2.8	-2.8	-2.8
10	Acetate	10	-2.8	-2.8	-2.7

Table S3. Docking score for Choline with respective to the ten poses and the binding affinity scores for replicate 1 (R1-SCORE), replicate 2 (R2-SCORE) and replicate 3 (R3-SCORE).

RANK	NAME	POSE	R1-SCORE	R2-SCORE	R3-SCORE
1	Choline	1	-3.5	-3.6	-3.5
2	Choline	2	-3.5	-3.5	-3.4
3	Choline	3	-3.5	-3.5	-3.4
4	Choline	4	-3.4	-3.5	-3.4
5	Choline	5	-3.4	-3.5	-3.3
6	Choline	6	-3.4	-3.3	-3.3
7	Choline	7	-3.3	-3.3	-3.3
8	Choline	8	-3.2	-3.2	-3.3
9	Choline	9	-3.2	-3.2	-3.3
10	Choline	10	-3.2	-3.2	-3.2

Table S4. Docking score for dihydrogenphosphate with respective to the ten poses and the binding affinity scores for replicate 1 (R1-SCORE), replicate 2 (R2-SCORE) and replicate 3 (R3-SCORE).

RANK	NAME	POSE	R1-SCORE	R2-SCORE	R3-SCORE
1	Dihydrogenphosphate	1	-4.1	-4.1	-4.1
2	Dihydrogenphosphate	2	-4.1	-4.1	-4.1
3	Dihydrogenphosphate	3	-3.9	-3.9	-3.9
4	Dihydrogenphosphate	4	-3.8	-3.8	-3.9
5	Dihydrogenphosphate	5	-3.8	-3.8	-3.9
6	Dihydrogenphosphate	6	-3.8	-3.8	-3.8
7	Dihydrogenphosphate	7	-3.8	-3.8	-3.8
8	Dihydrogenphosphate	8	-3.8	-3.8	-3.8
9	Dihydrogenphosphate	9	-3.7	-3.8	-3.8
10	Dihydrogenphosphate	10	-3.7	-3.6	-3.8

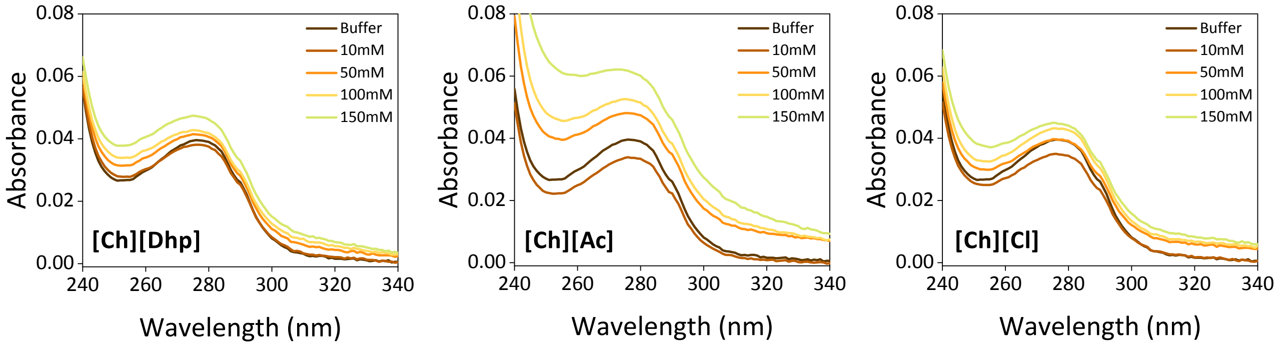

Figure S3. UV-vis spectra of *Rg*PAL in its aromatic region showing the effect of hydration on interactions with (a) [Ch][Cl], (b) [Ch][Ac] and (c) [Ch][Dhp] ILs.

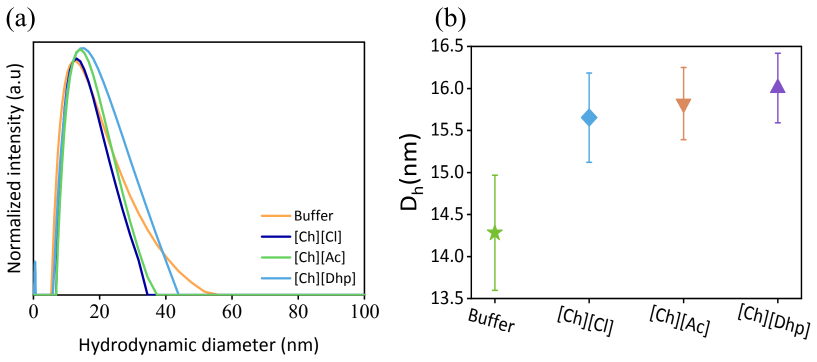

Figure S4. (a) Distribution curves of *Rg*PAL and (b) corresponding average hydrodynamic diameter in buffer and ILs media.

Table S5: The values of change in enthalpy (ΔH) and entropy (ΔS) of protein unfolding in buffer and IL+Tris-HCl systems.

	ΔHf_u (kcal mol^{-1})	ΔSf_u (kcal K^{-1} mol^{-1})
Buffer	24.53022 ± 0.12369	0.07351 ± 6.38E-4
[Ch][Cl]	19.07332 ± 0.24056	0.05615 ± 4.09E-4
[Ch][Ac]	20.28178 ± 0.23637	0.05972 ± 7.01E-5
[Ch][Dhp]	20.72396 ± 0.11458	0.06118 ± 5.72E-4
